# Resting vagally‐mediated heart rate variability in the laboratory is associated with momentary negative affect and emotion regulation in daily life

**DOI:** 10.1111/psyp.14668

**Published:** 2024-08-23

**Authors:** Lauren M. Bylsma, Kenneth G. DeMarree, Tierney P. McMahon, Juhyun Park, Kaitlyn M. Biehler, Kristin Naragon‐Gainey

**Affiliations:** ^1^ Department of Psychiatry and Psychology University of Pittsburgh Pittsburgh Pennsylvania USA; ^2^ Department of Psychology University at Buffalo, The State University of New York (SUNY) Buffalo New York USA; ^3^ School of Education and Social Policy Northwestern University Evanston Illinois USA; ^4^ Department of Psychology University of Toronto Scarborough Toronto Ontario Canada; ^5^ School of Psychological Science University of Western Australia Western Australia Australia

**Keywords:** affect, daily life, ecological momentary assessment, emotion regulation, heart rate variability, well‐being

## Abstract

Vagally‐mediated heart rate variability (vmHRV) is a physiological index reflecting parasympathetic activity that has been linked to emotion regulation (ER) capacity. However, very limited research has examined associations of physiological indices of regulation such as vmHRV with emotional functioning in daily life. The few studies that exist have small samples sizes and typically focus on only a narrow aspect of ER or emotional functioning. In this study, we examined associations between vmHRV assessed in the laboratory and emotional/mental health functioning in daily life using a 7‐day ecological momentary assessment design in 303 adult community participants. We hypothesized that higher resting vmHRV would be associated with higher positive affect (PA), lower negative affect (NA), less affective variability, greater well‐being, fewer dysphoria symptoms, greater use of engagement ER strategies, and less use of avoidance ER strategies, as assessed in daily life. Results revealed that higher resting vmHRV in the laboratory (as indexed by both high frequency heart rate variability, HF‐HRV, and the root mean of successive square deviations between heart beats, RMSSD) was significantly associated with less frequent use of avoidance ER strategies in daily life. Theoretical and clinical implications are discussed, including the association of vmHRV with negatively valenced, rather than positively valenced, daily life experiences.

## Abstract 2

Few studies have examined associations between laboratory autonomic nervous system activity and daily life emotional functioning. We report novel findings that higher resting parasympathetic activity in the lab is linked with lower negative affect and less reported use of avoidance as an emotion regulation strategy in daily life.

## 1 INTRODUCTION

Emotional processes are critical to daily life functioning, given that they facilitate adaptive responses to changing environmental circumstances and are associated with a range of important outcomes (e.g., well‐being, mental health, and social relationships; Smith & Lazarus, 1990). Upon experiencing an emotion, people may engage in emotion regulation (ER; i.e., implicit and explicit attempts to modify the type, intensity, or duration of an emotion), which may occur at various stages in the emotional response and may influence one or more emotion components (Gross, 2015, 1998). Given the large number of possible ER strategies individuals may use in daily life, some studies have tried to identify higher‐order groupings, which have generally converged on two broad domains: engagement/approach strategies (i.e., approaching and openly engaging with the emotion or situation eliciting the emotion, such as mindfulness or problem‐solving) and disengagement/avoidance strategies (i.e., attempting to divert attention from and/or failing to engage with some aspects of the emotion or situation eliciting the emotion, such as distraction or rumination) (e.g., Geisler et al., 2013; McMahon & Naragon‐Gainey, 2019). ER difficulties also have clinical significance as a transdiagnostic characteristic of many disorders (e.g., Aldao et al., 2010) and thus are a frequent treatment target in interventions (e.g., Gratz et al., 2015). As such, emotions and ER are closely yoked constructs that are important to consider together.

Emotions are multidimensional in nature, including experiential (self‐report), behavioral, physiological, cognitive, and neural components (see Bylsma, 2021). One key physiological marker relevant to emotional processes is vagally‐mediated heart rate variability (vmHRV), wherein higher levels of vmHRV (indicating greater sympathetic activity) have generally been linked to aspects of top‐down self‐regulation, including ER (Balzarotti et al., 2017), executive functioning, and effortful control (see Holzman & Bridgett, 2017 for a meta‐analysis). Indices of vmHRV include frequency domain (e.g., high frequency HRV, HF‐HRV), and time domain (e.g., Root Mean Successive Deviations, RMSSD), which are commonly used to quantify respiratory sinus arrhythmia (RSA; i.e., naturally occurring variation in heart rate linked to the breathing cycle via the vagus nerve) and reflect an individual's capacity to effectively regulate their emotions and flexibly respond to changing environmental conditions (Berntson et al., 1997). Specifically, vmHRV functions as a “vagal brake” that can suppress sympathetic activation when engaged or be withdrawn to allow an effective response to an environmental challenge, as needed for the situation (Appelhans & Luecken, 2006; Thayer et al., 2012; Thayer & Lane, 2000). Indices of vmHRV used to quantify RSA have demonstrated moderate stability over 18 months in both healthy and mood‐disordered adults (Seidman et al., 2023). Importantly, vmHRV can be increased via biofeedback techniques (e.g., Gevirtz, 2013), suggesting potential for incorporating vmHRV modification into treatment for emotion disorders that are characterized by ER difficulties, although prior biofeedback studies have focused primarily on increasing baseline levels of vmHRV.

Many studies have examined associations of vmHRV with subjective reports of emotional processes and clinical variables. Specifically, most of these studies have focused on associations between resting vmHRV assessed in the laboratory and retrospective trait self‐report measures of well‐being and ER across healthy, clinical, and community samples from adolescence through adulthood. Results have been relatively consistent in demonstrating associations between higher baseline vmHRV and trait measures reflecting greater well‐being, less severe depression, better affective functioning, less emotion dysregulation, and more adaptive ER (e.g., Bunford et al., 2017; Cai et al., 2019; Caldwell et al., 2023; Carnevali et al., 2018; Geisler et al., 2010; Koch et al., 2019; Licht et al., 2008; Williams et al., 2015).

Despite this large body of research from laboratory and retrospective self‐report measures, little is known about the associations between vmHRV and facets of emotional and mental health functioning as it is experienced *in real‐time in daily life*. Designs that measure these constructs in the context of daily life, such as ecological momentary assessment (EMA), may enhance ecological validity and clinical significance relative to retrospective measures, given that there are known recall biases associated with retrospective assessment of dynamic experiences (e.g., Gorin & Stone, 2001). Consistent with this idea, retrospective trait measures and momentary measures of ER in daily life (even those collected close in time) are generally unrelated or only weakly related (Koval et al., 2023; McMahon & Naragon‐Gainey, 2020). As such, scores on trait measures do not necessarily reflect momentary experience, highlighting the importance of specifically measuring emotion constructs in daily life. In addition, associations between vmHRV and emotional processes may vary with contexts that change over time and across situations (see Bylsma, 2021), and these fluctuations cannot be captured with retrospective trait assessments. Thus, EMA and similar methods may provide a clearer picture of the associations between emotional functioning in daily life and physiological processes.

A few recent studies have begun to examine associations between ambulatory vmHRV (i.e., assessed continuously in daily life) and emotional functioning in daily life, which also contribute to our understanding of how vmHRV and daily life emotional functioning are linked. For example, Määttänen et al. (2021) found that greater momentary vmHRV in an ambulatory study was associated with higher positive affect (PA) and lower negative affect (NA) in daily life. Further, Ottaviani et al. (2015) found that lower momentary vmHRV was associated with greater perseverative negative thinking (i.e., rumination and worry) in daily life for participants with depression and healthy controls, supporting the idea that higher vmHRV is linked to less use of avoidance ER strategies. Although momentary vmHRV associations with engagement ER strategies have not been assessed in daily life, laboratory tasks increasing mindfulness and interoceptive awareness (both reflecting greater engagement) can increase momentary vmHRV (Crowell et al., 2017; Gilbert & Gruber, 2014). Thus, these findings provide further support for the connection of vmHRV with daily life emotional functioning, though the results focus on momentary (within‐person) associations. Thus, it is not clear if they generalize to associations with individual differences in resting HRV.

Only a few studies have examined associations between resting vmHRV in the lab and emotional functioning in daily life, with most focusing on associations with affect and ER in particular. With regard to associations with resting laboratory vmHRV, Koval et al. (2013) found that higher vmHRV was associated with less instability of PA (i.e., using RMSSD) in daily life, but vmHRV was not clearly related to NA instability. Baseline vmHRV also predicted daily life ER strategy use in a 28‐day EMA study with college students, such that higher vmHRV was associated with less use of avoidance (disengagement) strategies (i.e., acceptance^1^ and avoidance) and greater seeking of social support in daily life. However, in this study, baseline vmHRV was not associated with use of engagement (approach) ER strategies (i.e., cognitive restructuring and problem solving) in daily life (Geisler et al., 2013). These results are consistent with a laboratory study that demonstrated that higher resting vmHRV was associated with less spontaneous use of avoidance in response to disgust‐eliciting emotional stimuli (Aldao et al., 2016). Finally, participants with higher laboratory resting vmHRV reported greater increases in daily PA over the course of 9 weeks (Kok & Fredrickson, 2010). The only study examining associations between vmHRV and daily life emotional functioning with EMA in a *clinical* sample (i.e., 162 adolescent youth [mean age = 12.03] receiving psychiatric treatment) demonstrated associations between resting vmHRV and greater dysregulated emotional experience (i.e., more variable emotional experience, as reflected by greater RMSSD) and self‐reported dysregulated behavior (e.g., emotional outbursts) in daily life (Byrd et al., 2022). However, no EMA studies have yet tested these associations in adult clinical samples.

### 1.1 The current study

Overall, several findings in the literature to date suggest that higher vmHRV is associated with more adaptive emotional functioning in daily life. However, the studies with adult samples cited previously are limited by modestly‐sized non‐clinical samples that consist primarily of undergraduate students (*N*s = 42–144), leading to concerns about limited power, replicability of findings, and generalizability to broader community or clinical samples. In addition, these studies did not assess associated clinical variables (e.g., well‐being, depression, other symptoms), and most narrowly focused on only a couple of different ER strategies or on effect (PA and/or NA). Given that affect, affective variability, ER, well‐being, and psychological symptoms are all closely related, studies that only include a few variables do not allow for robust conclusions about which variables are most strongly and specifically linked to vmHRV.

To provide a more comprehensive characterization of these associations, we measured resting vmHRV using both frequency and time domain measures (HF‐HRV and RMSSD) in the laboratory in a large and diverse community sample of adults. This was followed by a 7‐day ecological momentary assessment (EMA) protocol assessing affect (PA and NA), several clinical variables (i.e., well‐being, dysphoria symptoms), and six ER strategies in daily life that span engagement (i.e., reappraisal, savoring, mindfulness, decentering) and avoidance (i.e., distraction, rumination) strategies. We predicted that higher baseline vmHRV would be associated with higher PA, lower NA, less affective variability, greater well‐being, and less severe dysphoria symptoms in daily life. The ER strategies were reduced in an exploratory manner; and although factors could not be definitely specified a priori, we hypothesized that higher baseline vmHRV would be associated with greater use of the above engagement ER strategies, and less use of avoidance ER strategies in daily life.

## 2 METHOD

### 2.1 Transparency and openness

We report how we determined our sample size, all data exclusions, all manipulations, and all measures. The current study involves secondary analyses of a dataset collected to examine the impact of decentering on daily psychological experiences. All data and research materials are available at https://osf.io/gct7x/, and Mplus syntax for the current study is provided in the Online Supplement, Appendix A. The design and analysis of the larger study were preregistered at https://aspredicted.org/m2u6m.pdf, but the aims and analyses for the current paper were not preregistered. The findings from the larger study are reported in Naragon‐Gainey et al. (2023), and other portions of this dataset have also been reported elsewhere (Biehler & Naragon‐Gainey, 2022; DeMarree & Naragon‐Gainey, 2022; Park & Naragon‐Gainey, 2020; Sgherza et al., 2022). However, no previous use of this dataset has examined heart rate variability or its associations. The study was approved by the University at Buffalo Institutional Review Board (Protocol No. 00001194, “Decentering in Daily Life: Underlying Mechanisms and Impact on Well‐Being”).

### 2.2 Participants

The sample size was determined for the purposes of the larger study, with a target of at least 368 participants (more if funds allowed). An adult community sample of 379 individuals ages 18–65 was recruited for this study using online ads and flyers in the local community from 2017 to 2018. The recruiting strategy oversampled individuals who were seeking or receiving psychological treatment at the time of study enrollment (target = 50% of the sample) to increase the rates of clinically significant distress in the sample. Participants had to be English‐speaking and individuals who reported or showed evidence of a current cognitive impairment (e.g., dementia, intellectual disability, an active psychotic disorder, delirium) were not eligible for the study.

Of participants initially enrolled, one participant declined to participate in the vmHRV assessment and one was physically unable to wear the chest strap due to a medical condition. vmHRV could not be assessed for technical reasons (e.g., signal loss, excessive artifacts) in 11 participants. Of the remaining 366 participants who completed the vmHRV assessment, 2 participants were observed falling asleep during the vmHRV assessment and therefore were removed from analyses. Following vmHRV data processing, 4 people were excluded entirely from analyses and 29 people were excluded for one condition only (see Section 2.4 for details regarding the vmHRV conditions) due to poor quality signal, frequent artifacts, and/or short duration of useable (valid) data (i.e., <2 min). Of the 360 people with useable vmHRV data in one or both conditions, 11 participants who took statins and 5 participants who took corticosteroid medication were excluded from analyses, due to potential cardiovascular effects of these medications (Frasure‐Smith et al., 2009).

In addition, 30 of the remaining 344 participants did not enroll in the EMA protocol or completed fewer than 30% of reports,^2^ so they were also removed from analyses. Of the remaining 314 participants, 11 participants were removed from the sample who were vmHRV outliers (>3SDs from the mean), in addition to 7 outliers on only one of the vmHRV conditions. For the EMA data (6 reports per day for 7 days), individual reports were removed if they were completed outside the required 30‐min response window, or completed extremely quickly and without variability. After removing invalid reports, there were a total of 10,499 completed reports out of 12,726 possible reports. Thus, a mean of 82.5% of the reports (SD = 14.5%) were submitted and valid, ranging from 31% to 100% of reports across participants.

The final sample consisted of 303 participants with valid EMA data and useable data for at least one of the vmHRV conditions (68.0% female; mean age = 33.9, SD = 13.7, range = 18–65). In terms of race/ethnicity, 67.7% identified as White, 11.9% as Black or African American, 13.2% as Asian, 6.6% as more than one race, and 0.7% as Native American or Alaska Native. In addition, 6.6% identified as Hispanic or Latino/a. Of the participants, 52.2% had completed a 4‐year degree or higher degree, 28.1% with some college education, 13.2% with a 2‐year degree, and 6.6% with a high school diploma or some high school education. In regar employment, 33.7% were employed part‐time, 32.7% were full‐time students, 25.1% were unemployed, 25.1% were employed full‐time, and 5.9% were part‐time students (multiple responses possible). A majority of the participants (57.1%) were single (married = 19.8%; single but cohabitating with a partner = 12.5%; divorced = 9.6%; widowed = 1.0%). Most participants had a gross household income of less than $40,000 annually (<$10,000 = 30.7%; $10,000–$20,000 = 16.2%; $20,000–$40,000 = 23.8%; $40,000–$60,000 = 9.9%; $60,000–$80,000 = 6.3%; more than $80,000 = 13.2%).

Based upon a semi‐structured diagnostic interview, 42.2% of the sample met criteria for one or more emotional disorders, with the most common diagnoses being social anxiety disorder (25.4%), generalized anxiety disorder (20.5%), and unipolar depressive disorder (i.e., major depression and/or persistent depressive disorder; 11.4%). Interrater reliability was computed using a subset of independently rated interviews (*n* = 68). For disorders with 2 or more endorsements in the subsample, the mean kappa was .60, indicating moderate to substantial agreement on average (Cohen, 1960). Consistent with our sampling target, half of the sample (50.5%) either reported currently receiving therapy (35.6%) and/or taking psychiatric medication (32.3%).

### 2.3 Procedure

Initial eligibility screening was conducted via phone or email. Following informed consent at the 3 to 4‐h lab baseline assessment, individuals first completed a psychophysiological assessment of heart rate and vmHRV (see Section 2.4 for details), followed by computerized tasks and a battery of self‐report surveys on the computer, and then a semi‐structured clinical interview with a trained graduate student. Participants were compensated $50 for the baseline portion of the study.

Participants were then invited to enroll in a 7‐day follow‐up study where they completed 6 brief surveys daily using a smartphone, with the surveys sent at pseudo‐random times between 9 a.m. and 9 p.m. (i.e., randomly sent within each 2‐hour block, with at least 60 min between surveys). Study links to Qualtrics surveys were sent via text message using the SurveySignal system (Hofmann & Patel, 2015). Surveys began within 4 days of the baseline appointment and lasted for 7 days. Participants were shown how to complete the surveys and reviewed example items with the research assistant, who described several of the questions that were anticipated to be potentially unfamiliar or confusing.

Participants were asked to complete surveys within 30 min of receiving the survey link. Reminder text messages were sent after 20‐min if a participant had not yet completed the survey. Research assistants screened data daily and contacted participants if any problematic responding was identified. To encourage study engagement and to answer any questions, participants were also contacted via email 2 and 5 days into the follow‐up study. They were compensated $1.50 for each survey completed within the specified time frame, with an additional $15 bonus if at least 33 of 42 surveys were completed, for compensation of up to $78. Participants were also entered into a lottery to win one of four iPads, with the number of entries into the lottery equal to the number of valid surveys completed.

### 2.4 Measures

#### 2.4.1 Vagally‐mediated heart rate variability (vmHRV)

Participants completed the physiological recordings using a Polar H7 heart rate sensor, which is a chest strap electrocardiogram sensor that wirelessly transmits the R‐R interval (interbeat interval, IBI) time series data to a Bluetooth‐paired Polar V800 watch sampled at 1000 Hz (Polar Electro, Kempele, Finland). The Polar H7 has been validated as a reliable tool for the assessment of vmHRV (Hernández‐Vicente et al., 2021). There were two 5‐min heart rate recordings for all participants, which assessed heart rate under two resting baseline conditions. The first recording was the “vanilla” baseline condition (i.e., minimally demanding resting baseline task; Jennings et al., 1992), where participants were asked to attend to clips extracted from a travel video of Yosemite National Park (Expedia, 2016). The video was selected for its neutral content and emotional tone, lack of dialogue, and lack of social content. The second recording was a paced breathing condition that asked participants to breathe along with video and auditory guides, which controls for the potential influence of respiration rate on HF‐HRV (see Wilhelm et al., 2004). Specifically, an expanding figure on the screen and a high‐pitched tone were signals to inhale, whereas a contracting figure and low‐pitched tone were signals to exhale. If participants followed these cues, it would result in a consistent breath rate of 12 breaths per minute within and across participants, thereby controlling for the impact of respiration on HF‐HRV (Wilhelm et al., 2004). Research assistants monitored participants during physiological recordings to encourage them to follow the cues.

IBI time series data from these recordings were pre‐processed using ARTiiFACT 2.13 (Kaufmann et al., 2011). The first 30‐s of each recording was discarded due to excessive noise. Potential artifacts were identified using the Berntson detection method (Berntson et al., 1990). Each recording was manually inspected for both false positive and false negative artifact flags prior to processing. Identified artifacts were then corrected using cubic spline interpolation prior to calculation of indices. A window‐based linear detrending method was also used to account for longer term signal drifts (see Kaufmann et al., 2011). Heart rate (HR) was calculated as beats per minute (BPM). We computed both HF‐HRV, a frequency domain measure, and RMSSD of adjacent heart beats (R‐peaks), a time domain measure, which has been shown to be highly correlated with HF‐HRV (e.g., Allen et al., 2007). HF‐HRV was derived using spectral analysis with Fast Fourier Transformation (FFT) with a high frequency band window of 0.15–0.4 Hz, a Hanning window of 256 s and a 4 Hz spline interpolation rate, and the resultant HF power values were natural log‐transformed, consistent with the Task Force of the European Society of Cardiology and The North American Society of Pacing and Electrophysiology (1996) recommendations.

#### 2.4.2 Baseline covariates

Several baseline questions were included at the beginning of the self‐report measures that were considered as potential covariates in the current analyses. Specifically, participants reported their gender, age, any medications they used (which were examined to identify whether people were taking selective serotonin reuptake inhibitors [SSRIs; *n* = 74] and exclusionary medications [i.e., statins or corticosteroids]), and whether they had used any nicotine (*n* = 50) or caffeine (*n* = 229) in the previous 24 hours.

#### 2.4.3 Ecological momentary assessment (EMA)

Participants responded to each of a series of questions based on their experiences in the previous 30 min. All items used a 5‐point fully labeled scale (1 = *very slightly or not at all* to 5 = *extremely*), and multi‐item composites were summed. Constructs were randomly ordered for each survey with a random ordering of questions within each construct. Items were drawn from prior EMA studies where available, or adapted from baseline trait measures and theory; see Naragon‐Gainey et al. (2023) for further details regarding the sources and development of the EMA items.^3^ Convergent validity for the EMA items with validated baseline measures was generally acceptable and has been reported elsewhere (Naragon‐Gainey et al., 2023; Sgherza et al., 2022).

Table 1 provides the EMA items for each construct in the current study, as well as the between‐person internal consistencies (derived in multilevel structural equation modeling; see Geldhof et al., 2013) for any constructs with three or more items, and the between‐person item correlations for two‐item composites. The EMA survey also included items assessing each person's most bothersome symptom (idiographic symptoms), self‐control, and meta‐awareness. Self‐control, and meta‐awareness were not analyzed in the current study since we focused specifically on emotion and clinical variables, and the idiographic symptoms variable was excluded due to strong between‐person correlations with dysphoria and to improve model parsimony by not including too many predictors.

**TABLE 1:** Items, between‐person descriptive statistics, and between‐person zero‐order correlations for EMA assessment.

Construct	Items	Alpha/*r*	ICC
Negative affect (NA)	Recently I have felt… upset, irritable, afraid or anxious, sad	.92	.47
Positive affect (PA)	Recently I have felt… active, interested, excited, strong	.95	.51
Well‐being	How much meaning have you felt in life recently? To what extent have you felt alive & vital recently?	.89	.59
Dysphoria	Recently, I felt depressed; I had little interest in my usual hobbies and activities; I felt inadequate; I felt discouraged about things	.95	.61
Engagement ER	*Savoring*: Recently, I kept thinking about how happy and strong I felt	.72	.48
	*Mindfulness*: Recently, I have been focused on what is happening in the present moment		
	*Decentering*: Recently, I have been able to observe my thoughts and feelings without being drawn in		
Avoidance ER	*Distraction*: Recently, I did something or thought about something to distract myself from my feelings	.57	.48
	*Rumination*: Recently, I kept thinking about the causes or consequences of my negative feelings, how bad I felt		
Reappraisal	Recently, I changed the way I thought about what caused my feelings	—	.39

Between‐person *rs*	Mean (SD)	1	2	3	4	5	6	7	8	9
1. NA	7.0 (2.3)	1.00								
2. NA variability	2.3 (1.0)	0.57	1.00							
3. PA	9.8 (3.0)	−0.27	−0.17	1.00						
4. PA variability	2.7 (1.0)	−0.13	0.36	0.28	1.00					
5. Well‐being	5.4 (1.7)	−0.40	−0.23	0.89	0.28	1.00				
6. Dysphoria	6.9 (2.8)	0.83	0.46	−0.44	−0.22	−0.55	1.00			
7. Engagement ER	7.8 (1.8)	−0.24	−0.07	0.77	0.23	0.79	−0.37	1.00		
8. Avoidance ER	4.0 (1.7)	0.66	0.46	−0.12	−0.08	−0.19	0.60	−0.01	1.00	
9. Reappraisal	1.8 (1.2)	0.33	0.22	0.26	0.03	0.19	0.18	0.37	0.63	1.00

*Note*: All items were rated on a 1‐5 Likert‐type scale, so the maximum score for summed composites is 5 * number of items. Engagement and avoidance ER strategy composites were formed based results of a multilevel exploratory factor analyses (see Section 3 and Table 2). Between‐person alphas are presented for composites with three or more items (i.e., the minimum number of items needed to compute alpha), and between‐person item correlations are shown for two‐item composites. Correlations > |.09| are significant at *p* < .05.

Abbreviation: ER, emotion regulation.

### 2.5 Data analysis

Multilevel structural equation modeling (MSEM) in Mplus 8.7 was used to account for the nested structure of the EMA data, as MSEM isolates between‐person variance in analyses so that it is independent of variability within individuals across reports. We used robust maximum likelihood estimation to appropriately account for missing data and non‐normality.

Given the relatively large number of ER strategies assessed with single items and prior evidence for underlying strategy factors in daily life (McMahon & Naragon‐Gainey, 2019), we first conducted a multilevel exploratory factor analysis to attempt to reduce the data. The within‐person variances of each variable were allowed to freely vary and covary, and between‐person solutions with one to three factors were examined. To determine the optimal factor solution, we considered the model fit and interpretability. Model fit was evaluated using the following guidelines: the comparative fit index (CFI) should be near .95 or above for excellent fit and .90 to .95 for good fit (Hu & Bentler, 1999), the root mean square error of approximation (RMSEA) should be at or below .06 (Hu & Bentler, 1999), and the standardized root mean square residual (SRMR) should be at or below .08 (Browne & Cudeck, 1992).

To address the primary study aims, multilevel models were run predicting vmHRV from the between‐person variance of daily life emotion and clinical variables and from positive and NA variability (indexed as the standard deviation of PA or NA scores for each person across their EMA reports). Potential covariates (gender, age, SSRI use, caffeine use, nicotine use) were included in the regression analyses if they had a significant zero‐order association with vmHRV (e.g., Kemp et al., 2016; O'Regan et al., 2015). We then ran regression models predicting vmHRV (RMSSD, HF‐HRV) from significantly associated covariates and daily life variables. Predictors were allowed to freely (co)vary at the within‐person level to account for intraindividual variability over time.^4^

## 3 RESULTS

### 3.1 Preliminary analyses and descriptive statistics

Before conducting hypotheses tests, we first examined variable descriptive statistics and correlations, as well as the factor structure of the ER strategies. vmHRV and heart rate (HR) mean values were within expected ranges. The mean vmHRV (RMSSD) for the resting vanilla baseline condition (*n* = 292) was 29.28 (SD =20.4, range = 2.8–101.9), mean vmHRV (HF‐HRV) was 5.51 (SD = 1.5, range = .09–8.7), and mean HR was 79.3 bpm (SD = 12.0, range = 54.7–119.4). Mean vmHRV (RMSSD) in the resting paced breathing condition (*n* = 288) was 29.4 (SD = 19.1; range = 3.3–99.8), mean vmHRV (HF‐HRV) was 5.94 (SD = 1.5, range = .9–8.9), and mean HR was 80.8 bpm (SD = 12.2, range = 43.6–127.5). For RMSSD, the means in the two resting baseline conditions did not significantly differ from one another (Wald test (1) = .94, *p* = .33), although they did significantly differ for HF‐HRV (Wald test (1) = 70.36, *p* < .001). The vmHRV means for the two resting baselines were also highly correlated for both RMSSD and HF‐HRV (*r*s = .86–.88), suggesting that the influence of respiration rate on resting vmHRV was negligible in our sample. Similarly, the correlation between vmHRV indexed with RMSSD and HF‐HRV in both conditions was very strong (*r*s = .86).

Results of the multilevel exploratory factor analysis of ER strategies (i.e., rumination, distraction, savoring, reappraisal, mindfulness, decentering) indicated that a one‐factor solution did not fit adequately based upon SRMR (CFI = .97, RMSEA = .01, SRMR between = .18), and the three‐factor solution did not converge on a proper solution. However, the two‐factor solution fit the data very well (*χ* ^*2*^(4) = 10.45, *p* = .034, CFI = 1.0, RMSEA = .00, SRMR between = .04); see Table 2 for the standardized factor loadings. The two‐factor solution was interpretable as avoidance strategies (marked by rumination and distraction; loadings = .66–.83) and engagement strategies (marked by savoring, mindfulness, and decentering; loadings = .64–.74), and these factors were uncorrelated (*r* = .02). However, reappraisal loaded substantially and positively on both factors.^5^ These results generally align with the between‐person structure found in McMahon and Naragon‐Gainey (2019), consisting of engagement and avoidance strategy factors, with reappraisal loading positively on both in this study as well. Thus, for all subsequent analyses, we analyzed avoidance (rumination and distraction) and engagement (savoring, mindfulness, and decentering) composites. However, reappraisal was analyzed separately as a single item since it was not a specific indicator of either factor.

**TABLE 2:** Standardized between‐person factor loadings for ER strategies.

	Avoidance	Engagement
Rumination	**.83** *	−.17*
Distraction	**.66** *	.00
Reappraisal	**.80** *	**.37** *
Savoring	.18*	**.70** *
Mindfulness	−.08	.**64** *
Decentering	−.00	**.74** *

*Note*: Values >0.30 are shown in bold. Indicators were allowed to freely vary and covary at the within‐person level.

* *p* < .05.

Descriptive statistics of the daily life emotion and clinical variables are presented in Table 1, including between‐person means, standard deviations, and correlations, as well as intraclass correlations (ICCs). ICCs ranged from .39 to .61, indicating substantial variability between‐persons for the current analyses. In addition, the between‐person variance of NA, dysphoria, and avoidance strategies were all strongly correlated (*r*s = .59 to .83), as were PA, well‐being, and engagement strategies (*r*s = .77 to .89). Mean levels of NA and NA variability were also strongly correlated (*r* = .57).

Table 3 displays the correlations of resting vmHRV (indexed with RMSSD and HF‐HRV) scores during the vanilla and paced breathing resting baseline conditions with the potential covariates and between‐person variance of the EMA variables. For the covariates, greater age (*r*s = −.38 to −.53) and SSRI use (*r*s = −.11 to −.16) were associated with lower vmHRV in both conditions. Higher nicotine use was also associated with higher vmHRV in the vanilla baseline condition (*r*s = .11–.15), and more caffeine use was associated with higher vmHRV in the paced breathing baseline condition indexed with HF‐HRV (*r* = −.16). With regard to emotion and clinical variables in daily life, less use of avoidance ER strategies (i.e., rumination and distraction) in daily life was significantly associated with higher vmHRV in the paced breathing condition only (*r* = −.13). No other zero‐order associations with vmHRV reached conventional significance thresholds, and the effect sizes were small (*r*s = |.01| to |.12|).

**TABLE 3:** Correlations of resting vmHRV with covariates (top) and between‐person variance of daily life emotional and clinical variables (bottom).

	Vanilla Baseline		Paced Breathing Baseline	
	RMSSD	HF‐HRV	RMSSD	HF‐HRV
Covariates				
Gender	−.03	−.03	−.03	−.01
Age	−.41***	−.53***	−.38***	−.53***
Nicotine use	.11*	.15*	.08	.10
Caffeine use	.07	.07	.10	.14**
SSRI use	−.13**	−.11	−.15**	−.16**
Daily life variables				
NA	−.06	−.06	−.08	−.09
NA variability	−.05	−.06	−.07	−.06
PA	.07	.05	.06	.06
PA variability	.06	.05	.03	.04
Well‐being	.05	.04	.05	.06
Dysphoria	−.04	−.07	−.09	−.08
Engagement ER	.02	.01	.02	.04
Avoidance ER	−.10	−.12	−.13*	−.12
Reappraisal	−.06	−.09	−.04	−.05

*Note*: *r* between vanilla and paced breathing baseline conditions = .88 for RMSSD and .86 for HF‐HRV. *r* between RMSSD and HF‐HRV = .86 for vanilla condition and .86 for paced condition. Gender was coded as male = 1 and female = 2.

Abbreviations: ER, emotion regulation; HF‐HRV, high frequency heart rate variability; NA, negative affect; PA, positive affect; RMSSD, root mean square of successive differences; SSRI, selective serotonin reuptake inhibitor.

* *p* < .05.

** *p* < .01.

*** *p* < .001.

### 3.2 HRV as predicted by daily life emotion and clinical variables

Next, we conducted multilevel models predicting vmHRV from the between‐person variance of the daily life variables and from PA and NA variability. Because the resting vmHRV scores for the vanilla and paced breathing resting baseline conditions were so highly correlated as to be redundant (*r* = .86), we conducted the regression analyses using the paced breathing resting baseline only. The paced breathing condition was selected because it controls for the effects of respiration on vmHRV and therefore may better isolate the process of interest relative to the vanilla condition, and it tended to show slightly stronger effect sizes with daily life variables (see Table 3). Results from multilevel regressions predicting resting vmHRV from daily life emotion and clinical variables, age, SSRI use, and caffeine use (HF‐HRV only) are shown in Table 4. Age was a significant inverse predictor of vmHRV (*p*s < .001), but SSRI use and caffeine use were not associated with vmHRV (*p*s > .10). After accounting for these covariates, less use of avoidance ER strategies (i.e., rumination and distraction) was associated with higher vmHRV (*p*s < .05) in both models. In addition, in the HF‐HRV model only, lower NA and *greater* dysphoria symptoms were associated with higher vmHRV (*p*s < .01).

**TABLE 4:** Multilevel regressions predicting baseline vmHRV from daily life emotion and clinical variables and covariates.

Predictor variable	RMSSD			HF‐HRV		
	*β*	SE	*p*	*β*	SE	*p*
Covariates						
Age	−.38***	.04	.000	−.52***	.04	.000
SSRI use	−.09	.05	.101	−.07	.05	.170
Caffeine use	—	—	—	.04	.04	.272
Daily life variables						
NA	−.15	.08	.069	−.21**	.08	.009
NA variability	.04	.08	.641	.04	.07	.558
PA	.10	.10	.300	.06	.09	.474
PA variability	.00	.07	.948	.02	.06	.762
Well‐being	−.05	.11	.646	−.03	.09	.718
Dysphoria	.16	.10	.111	.23**	.08	.004
Engagement ER	.02	.10	.848	.09	.09	.312
Avoidance ER	−.19**	.07	.006	−.16*	.07	.016
Reappraisal	.04	.07	.565	.01	.06	.915

*Note*: *N* = 288. Standardized regression coefficients and standard errors are shown. Baseline vmHRV (RMSSD, HF‐HRV) values from the paced breathing condition were used in the analyses depicted here.

Abbreviations: ER, emotion regulation; HF‐HRV, high frequency heart rate variability; NA, negative affect; PA, positive affect; RMSSD, root mean square of successive differences; SSRI, selective serotonin reuptake inhibitor.

* *p* < .05.

** *p* < .01.

*** *p* < .001.

## 4 DISCUSSION

We aimed to comprehensively examine associations between resting vmHRV measured in the laboratory with affect, affective variability, well‐being, dysphoria symptoms, and ER strategy use in daily life in a large and diverse community sample of adults. Overall, consistent with some of our predictions, we found that higher resting vmHRV was significantly associated with less frequent use of ER strategies characterized by avoidance/disengagement in daily life (i.e., rumination and distraction), which is generally aligned with the limited prior literature in this area (e.g., Geisler et al., 2013; Määttänen et al., 2021). These findings were consistent for both vmHRV indices, RMSSD and HF‐HRV, whereas lower NA was associated with higher vmHRV for HF‐HRV only. These findings increase confidence in the ecological validity and generalizability of prior vmHRV associations with trait NA and avoidance strategies, using assessments that occur in real‐life daily contexts and do not depend on lengthy retrospective recall. The particularly robust association with avoidance strategies in both vmHRV indices may support the interpretation of vmHRV as primarily and specifically an index of regulation or top‐down control (e.g., Holzman & Bridgett, 2017), rather than of emotional reactivity or affective tendencies more broadly, which may help clarify prior heterogeneous findings in the literature. These findings are also consistent with fear‐avoidance models linking heightened physiological arousal (i.e., greater sympathetic relative to parasympathetic activity) with increased avoidance behavior (see Grisham et al., 2015; Norton & Asmundson, 2003). However, as we did not measure sympathetic activity in this study, future studies should examine both parasympathetic and sympathetic activity simultaneously to provide more support for this idea.

In contrast to prior literature with undergraduate (Koval et al., 2013) and adolescent samples (Byrd et al., 2022), we did not find significant associations with other constructs examined, including affective variability. However, given strong predictive effects of age for vmHRV in the current study (see also Garavaglia et al., 2021), it may be difficult to compare findings in samples with very different age ranges and means. It is possible that associations between vmHRV and affective variability may be stronger in younger individuals, particularly during early adolescence when variability of both positive and negative emotions peaks (see Reitsema et al., 2022, for review). Given the breadth of affective and mental health/well‐being constructs that are related to vmHRV, studies such as this that examine a range of relevant constructs and their unique predictive variance may help clarify the psychological interpretation of vmHRV and its nomological network with emotion variables.

It was somewhat surprising that dysphoria was not predictive of vmHRV, given that lower vmHRV is associated with depression in numerous studies (e.g., Koch et al., 2019; Licht et al., 2008). While we found no association between dysphoria and RMSSD, there was a paradoxical effect of greater dysphoria associated with higher HF‐HRV. This reflects a suppressor effect, given that the zero‐order association of dysphoria with HF‐HRV was negative (albeit non‐significant), but the association became positive with the addition of other predictors in the regression model. Such a finding makes sense given the very strong association of dysphoria with other predictors in the model (*r* with NA = .83), which means that the unique variance of dysphoria in the regression model was likely quite small. Consistent with this, a post hoc analysis revealed that dysphoria was not a significant predictor of HF‐HRV when NA was removed from the model. Thus, the finding involving dysphoria predicting higher HF‐HRV should be interpreted with caution. It may also be that prior relationships between vmHRV and depression in the literature are driven more by somatic symptoms, such as sleep difficulties (Bylsma et al., 2014; Werner et al., 2017), as the dysphoria items in the current study only assessed emotional (depressed mood, anhedonia) and cognitive/motivational (felt inadequate, felt discouraged) symptoms of depression.

It was also noteworthy that none of the positively valenced or adaptive constructs in the current study (i.e., PA, well‐being, reappraisal, and engagement strategies of mindfulness, decentering, and savoring) were associated with resting vmHRV. Rather, all observed associations were with negatively valenced or maladaptive constructs (i.e., NA, avoidance ER strategies; but see above regarding null findings for dysphoria). This is unexpected, given prior evidence of associations with positively valenced constructs using retrospective assessment of psychological variables (e.g., Geisler et al., 2010; Kok & Fredrickson, 2010), and in some cases associations were found *only* for positively valenced, but not negatively valenced, constructs (e.g., Koval et al., 2013; Oveis et al., 2009; Wang et al., 2013). However, as noted by Sloan et al. (2017), most of these studies were in small samples of about 100 or fewer participants, generally using undergraduate participants with limited age ranges. In a study using trait questionnaires and resting vmHRV with a well‐powered nationally representative sample of nearly 1000 participants, Sloan et al. (2017) reported a significant association of vmHRV with lower trait NA, but not with trait PA or a range of well‐being measures. Similarly, our finding of daily life associations with avoidance but not engagement in ER strategies is consistent with the results of Geisler et al. (2013). While further research is needed— particularly regarding daily life experiences— our study and some other prior findings suggest that higher vmHRV may be more closely linked to the reduced experience of NA and strategies that tend to increase it, as opposed to greater PA and related cognitions and behaviors. If replicated, these findings may have potential clinical implications for the identification and targeting of avoidance ER processes in individuals with mental health disorders. For example, just‐in‐time adaptive interventions (JITAIs) could be developed to target vulnerable periods of low vmHRV in daily life as critical intervention points to reduce subsequent avoidant ER in response to NA in order to mitigate the potential maladaptive outcomes of this strategy.

### 4.1 Strengths, limitations, constraints on generality, and future directions

This study is the first to comprehensively examine associations between baseline vmHRV and emotional functioning in daily life in a large and diverse community sample, with a wide range of ages represented across adulthood. However, there are several limitations. First, regarding *constraints on generality*, although we over‐sampled for treatment‐seeking adults and about half of the sample met current criteria for a disorder, the remainder of the sample were non‐clinical community members. As such, it is possible that associations may be different in a sample who all had clinically significant symptoms or were more homogenous with regard to diagnoses (e.g., all had major depressive disorder). In addition, we tested a single geographical location in the Northeastern US, and women and White participants were over‐represented. Second, although we assessed a broader range of constructs in daily life than in prior studies, we still only included a relatively small number of possible ER strategies and psychological symptoms, and the EMA study design necessitated that only brief measures of each construct could be included. As such, future research should build on this to increase the breadth of constructs assessed, allowing for stronger conclusions about the associations of vmHRV with emotion‐related experiences in daily life. Third, we were not able to assess for all possible covariates that may contribute to individual differences in vmHRV, such as body mass index or physical activity levels.

Future studies could also extend this work by examining momentary associations between these constructs and HRV, while also incorporating situational context or emotional reactivity to specific daily life situations. For example, there is evidence that social context may moderate associations between vmHRV and ER (see Gerteis & Schwerdtfeger, 2016). It would also be beneficial to examine the influence of context in daily life situations that trigger emotional reactions, to better characterize associations between vmHRV and daily life emotional functioning. In addition, measuring ambulatory vmHRV in daily life would allow for within‐person tests of the associations of vmHRV with emotional experiences, including tests of directionality and temporal precedence. Overall, though further research is required to replicate and extend these results, the current study provides support for the relevance of resting vmHRV to daily life emotional processes in a large community sample, and clarifies that it may be primarily associated with momentary use of avoidance ER strategies.

## AUTHOR CONTRIBUTIONS

**Lauren M. Bylsma:** Conceptualization; formal analysis; methodology; supervision; writing – original draft; writing – review and editing. **Kenneth G. DeMarree:** Conceptualization; data curation; funding acquisition; methodology; project administration; supervision; writing – original draft; writing – review and editing. **Tierney P. McMahon:** Data curation; investigation; writing – review and editing. **Juhyun Park:** Data curation; investigation; writing – review and editing. **Kaitlyn M. Biehler:** Data curation; investigation; writing – review and editing. **Kristin Naragon‐Gainey:** Conceptualization; data curation; formal analysis; funding acquisition; methodology; project administration; resources; supervision; writing – original draft; writing – review and editing.

## FUNDING INFORMATION

This research was supported by National Center for Complementary and Integrative Health/National Institutes of Health Grant R21AT009470, “Decentering in Daily Life: Underlying Mechanisms and Impact on Well‐Being,” to K. Naragon‐Gainey and K. G. DeMarree. The content is solely the responsibility of the authors and does not necessarily represent the official views of the National Institutes of Health. The authors have no conflicts of interest to report. All data and research materials are available at https://osf.io/gct7x/.

## Supporting information


**Appendix A:** Mplus Syntax for Analyses.

## Data Availability

All data and research materials are available at https://osf.io/gct7x/.

## Footnotes

^1^
Note that acceptance is often considered an approach or engagement ER strategy (e.g., McMahon & Naragon‐Gainey, 2019), rather than an avoidance strategy as it was in this study. However, the item used to assess acceptance in Geisler et al. (“I thought I had to accept the situation”) suggests resignation or a sense of “coerced” acceptance of a situation that is different from operationalizations of acceptance as a freely chosen openness to one's current thoughts and emotions, and therefore the wording in that study may align more with avoidance strategies.

^2^
The final sample did not differ from the participants who were excluded from EMA analyses in terms of sex, age, ethnicity, education level, marital status, and employment status (*p*s > .05). However, there were significant differences for race and household income between the two groups (*p*s < .05).

^3^
Note that four decentering items were included in data collection to model two components of decentering: disidentification from internal experiences and reduced reactivity to thoughts (Naragon‐Gainey et al., 2023). We focus in the current study on the disidentification from internal experiences component because it is conceptually most strongly related to ER attempts, and it is more distinct from perseverative negative thoughts (e.g., rumination). One of the disidentification from internal experiences items performed poorly (see Naragon‐Gainey et al., 2023), so we selected the better performing single item as the measure of decentering in the current study.

^4^
Note that our assessment of vmHRV and daily life variables is not strongly temporally ordered, given that they were measured days apart and the between‐person variance of daily life variables was used in analyses. As such, this study primarily tests associations, rather than prediction, and vmHRV could be modeled as the independent variable or outcome. We chose to model vmHRV as the outcome variable in this case because we could then run follow‐up multivariate analyses controlling for shared variance between significant daily life variables in association with HRV, which would not be possible if vmHRV were the independent variable.

^5^
See Sgherza et al. (2022) for discussion of the properties and performance of the reappraisal item in this dataset, which may be partly due to varied interpretations of the item and/or relatively low endorsement rates.
