# Supplementary material for: Resting vagally‐mediated heart rate variability in the laboratory is associated with momentary negative affect and emotion regulation in daily life
Source: Psychophysiology. 2024 Aug 23;61(12):e14668. doi: 10.1111/psyp.14668 (PMC11579239; doi:10.1111/psyp.14668)
Supplement: Supplementary file 1 — Appendix A: Mplus Syntax for Analyses. [file PSYP-61-e14668-s001.docx]

**Online Supplement**

**Appendix A: Mplus Syntax for Analyses**

***Multilevel Exploratory Factor Analysis of Emotion Regulation Strategies***

VARIABLE:

USEVAR ARE Brood Distract Savor Reapprais Mindful Decent1;

CLUSTER = ID;

MISSING = ALL (-999, -999.0, -999.00);

USEOBS is (PctEMAComp GE .30 AND MedControl LE 1 AND ID NE 262 and ID NE

363 AND 326 AND ID NE 431 AND ID NE 112 AND ID NE 191 AND ID NE 222

AND ID NE 275 AND ID NE 313 and ID NE 341AND ID NE 430 and ID NE 486 and ID

NE 512 and ID NE 542 and ID NE 184 and ID NE 268 and ID NE 277 and ID NE 318

and ID NE 319 and ID NE 365 and ID NE 395 and ID NE 496 and ID NE 534 and ID NE

538 and ID NE 551 and ID NE 442 and ID NE 504 and ID NE 507 and ID NE 568 and ID

NE 139 and ID NE 413 and ID NE 441 and ID NE 497 and ID NE 299);

ANALYSIS:

TYPE = twolevel efa 1 3 UW 1 3 UB;

ESTIMATOR = MLR;

***Multilevel Regression Analyses Predicting Baseline HRV from Daily Life Emotional and Clinical Variables***

*Note:* This syntax is for RMSSD, but the same analysis was also run using HF-HRV instead of RMSSD.

VARIABLE:

USEVAR ARE PacedHRV age NA NAsd PA PAsd wellbeing dysphoria reapprais

approach avoid SSRI;

BETWEEN is PacedHRV age NAsd PAsd SSRI;

CLUSTER = ID;

MISSING = ALL (-999, -999.0, -999.00);

USEOBS is (PctEMAComp GE .30 AND MedControl LE 1 AND ID NE 262 and ID NE 363 AND 326 AND ID NE 431 AND ID NE 112 AND ID NE 191 AND ID NE 222 AND ID NE 275 AND ID NE 313 and ID NE 341AND ID NE 430 and ID NE 486 and ID NE 512 and ID NE 542 and ID NE 184 and ID NE 268 and ID NE 277 and ID NE 318 and ID NE 319 and ID NE 365 and ID NE 395 and ID NE 496 and ID NE 534 and ID NE 538 and ID NE 551 and ID NE 442 and ID NE 504 and ID NE 507 and ID NE 568 and ID NE 139 and ID NE 413 and ID NE 441 and ID NE 497 and ID NE 299);

DEFINE:

IF (MedControl==1) THEN SSRI=1;

IF (MedControl==0 or MedControl==2 or MedControl==3) THEN SSRI=0;

Avoid = Brood + Distract;

Approach = Savor + Mindful + Decent1;

ANALYSIS:

TYPE = twolevel;

ESTIMATOR = MLR;

MODEL:

%WITHIN%

NA; PA; wellbeing; dysphoria; reapprais; approach; avoid;

NA with PA wellbeing dysphoria reapprais approach avoid;

PA with wellbeing dysphoria reapprais approach avoid;

wellbeing with dysphoria reapprais approach avoid;

dysphoria with reapprais approach avoid;

reapprais with approach avoid;

approach with avoid;

%BETWEEN%

PacedHRV ON NA NAsd PA PAsd wellbeing dysphoria approach avoid reapprais age SSRI;

OUTPUT: STDYX; SAMPSTAT; MODINDICES;
